# Improving mental health care for unaccompanied young refugees through a stepped-care approach versus usual care+: study protocol of a cluster randomized controlled hybrid effectiveness implementation trial

**DOI:** 10.1186/s13063-020-04922-x

**Published:** 2020-12-09

**Authors:** Rita Rosner, Cedric Sachser, Fabienne Hornfeck, Reinhold Kilian, Heinz Kindler, Rainer Muche, Lauritz Rudolf Floribert Müller, Jonathan Thielemann, Tamara Waldmann, Ute Ziegenhain, Johanna Unterhitzenberger, Elisa Pfeiffer

**Affiliations:** 1 Department of Psychology, Catholic University Eichstätt-Ingolstadt, Ostenstr. 26, 85071 Eichstätt, Germany; 2 Clinic for Child and Adolescent Psychiatry/Psychotherapy, Ulm University, Steinhövelstr. 1, 89075 Ulm, Germany; 3 German Youth Institute, Nockherstr. 2, 81541 Munich, Germany; 4 Clinic for Psychiatry and Psychotherapy II, Ulm University, Ludwig-Heilmeyer-Str., 89312 Günzburg, Germany; 5 Institute of Epidemiology and Medical Biometry, Ulm University, Schwabstr. 13, 89075 Ulm, Germany

**Keywords:** Stepped care, Cluster randomized trial, Dissemination, Refugee minors, Posttraumatic stress, Interpreters, Trauma-informed care, Healthcare costs

## Abstract

**Background:**

More than half of the unaccompanied young refugees (UYRs) resettled in Europe report elevated levels of posttraumatic stress symptoms (PTSS) and comorbid symptoms. Earlier studies have highlighted the effectiveness of the trauma-focused preventive group intervention “Mein Weg” (English “My Way”), and the feasibility of trauma-focused cognitive behavioral therapy (TF-CBT) for UYRs. Both interventions are deemed to be empirically supported treatments (ESTs). However, UYRs seldom receive ESTs or, in fact, any treatment at all. In view of the high need and the limited treatment resources available, a stepped-care approach is indicated but has not been evaluated so far. The purpose of this trial is to compare the stepped-care approach BETTER CARE with usual care enhanced with screening and indication (usual care+).

**Methods:**

In a cluster randomized controlled trial involving *N* = 540 UYRs living in up to *N* = 54 child and youth welfare service (CYWS) facilities, BETTER CARE will be compared with usual care+. We will randomize clusters comprising a CYWS facility with at least one eligible psychotherapist. BETTER CARE consists of step (1) screening and indication and either step (2) preventive trauma-focused group intervention “Mein Weg” delivered by trained CYWS staff or step (3) TF-CBT delivered by trained community therapists and supported by trained translators if necessary. Participants will be assessed 6 and 12 months after randomization. The primary outcome is the severity of PTSS after 12 months. Secondary outcomes are depressive and anxiety symptoms, quality of life, and proxy reported PTSS. Furthermore, drug use, health costs, benefits, and long-term effects on integration/acculturation will be assessed.

**Discussion:**

The trial will directly integrate a stepped-care approach into existing structures of the German child welfare and (mental) health system. It could, therefore, serve as a blueprint for how to implement ESTs for UYRs. If successful, screening, prevention, and intervention will be sustainably implemented in CYWS in southern Germany.

**Trial registration:**

German Clinical Trials Register DRKS00017453. Registered on 11 December 2019.

**Supplementary information:**

The online version contains supplementary material available at 10.1186/s13063-020-04922-x.

## Background

Refugee minors often arrive in European host countries without their parents or other caregivers. In Germany, unaccompanied refugee minors have the possibility to remain in the care of child and youth welfare services (CYWS) up to the age of 21. We, therefore, use the terms minors and young adults when referring to unaccompanied young refugees (UYRs). However, there are reports that CYWS staff have been confronted with major challenges, for instance, limited professional experience and insufficient training [1]. A recent cross-sectional study showed that UYRs in southern Germany had experienced a high number of traumatic events, and presented elevated levels of posttraumatic stress symptoms (PTSS), depression, and anxiety [2]. Symptoms, especially within the re-experiencing cluster, seem to be central in this population [3], and they differ in this respect from other traumatized children and adolescent samples [4]. These findings are in line with several studies from other European countries reporting high PTSS rates from 20% [5] to 76% [6]. On average, symptoms tend to be stable over time [7] even though another recent study by Müller et al. [8] indicated a significant reduction in PTSS severity after 12 months. However, the rates for clinically relevant PTSS (38%) were still high. Additionally, UYRs often suffer from ongoing stressors in their host countries, so-called post-migration stressors, such as worries about their families or acculturation distress [9].

School- and community-based interventions in a group setting are one way of meeting the high demand for mental health care in vulnerable populations [10]. They offer low-threshold support even for individuals with sub-clinical PTSS. The 6-session trauma-focused preventive intervention “Mein Weg” (English “My Way” [11]) enables social workers in CYWS facilities to support UYRs with PTSS and related difficulties. In their randomized controlled trial with *N* = 99 UYRs, Pfeiffer and colleagues [12] demonstrated the effectiveness and sustainability of the trauma-focused group program [13]. The intervention manual, which is based on trauma-focused cognitive behavioral therapy principles, was specifically developed for UYRs in collaboration with CYWS facilities, for instance, by using pictures instead of writing, thus making them more accessible to UYRs with limited German language or reading skills [11]. Despite a number of publications on “Mein Weg” studies [12–15], it has not been evaluated as a preventive component within a stepped-care approach.

For UYRs who meet the diagnostic criteria of posttraumatic stress disorder (PTSD), an evidence-based, individual trauma-focused treatment by a mental health care professional is indicated [16]. Trauma-focused cognitive behavioral therapy (TF-CBT) [17] is one of the treatments with the most empirical support in western and non-western cultures [18]. As it includes a caregiver in treatment, it is specifically suited to improving social support outside of therapy for UYRs living in CYWS facilities. Unterhitzenberger and colleagues [19] enhanced the cultural sensitivity of TF-CBT for UYRs and verified its feasibility in an uncontrolled trial. In fact, TF-CBT is the only individual treatment that has been evaluated with UYRs up to now. Evidence in the context of an RCT and a stepped-care approach is lacking. TF-CBT has proven its effectiveness in the German health care system within an RCT in eight outpatient clinics [20]. The dissemination and sustainable implementation of TF-CBT in German routine care are now necessary.

**Fig. 1:**
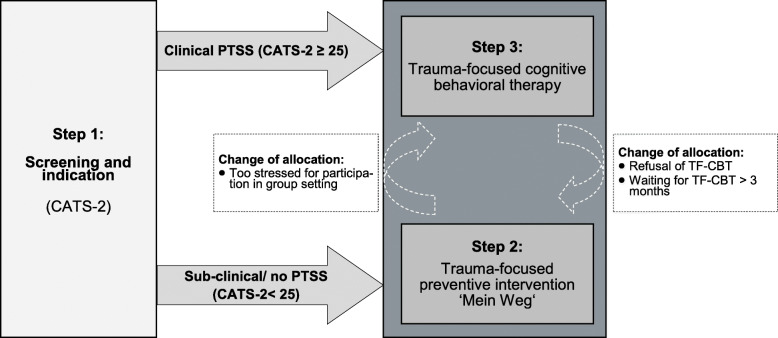
The stepped-care approach BETTER CARE. CATS-2 = Child and Adolescent Trauma Screen 2; TF-CBT = trauma-focused cognitive behavioral therapy

Based on the authors’ preliminary work on the mental health of minor refugees, the effectiveness of “Mein Weg,” and the feasibility of TF-CBT for UYRs, the intervention to be evaluated is a stepped-care approach consisting of three main components: screening, preventive group intervention, and individual treatment. We call this approach BETTER CARE (see Fig. 1). Cascade, tier, or stepped-care models to treat traumatized refugees have been suggested before [21, 22] but have rarely been evaluated. Not only is there a lack of information on their effectiveness, but also on their implementation and health economic evaluation. The latter is especially relevant, as there are no data on the cost-effectiveness of PTSD treatments for UYRs in Germany and a stepped-care approach is supposed to be especially needs-oriented. BETTER CARE will be implemented in the routine care of CYWS and health services in Germany. Some studies have investigated general attitudes of CYWS staff towards the mental health treatment of their clients. Results show that CYWS staff perceive mental health treatment as necessary but rate cooperation between systems as difficult [23]. A systematic analysis of successful factors on the institutional level (e.g., motivation, capacity of facilities, collaboration with mental health sector) is necessary to ensure the implementation of BETTER CARE [24].

## Methods

### Aims

We aim to implement and evaluate a screen and stepped-care approach for UYRs by providing empirically supported treatments (ESTs) through dissemination and implementation. The objectives are as follows:

1. To compare BETTER CARE with a control group (usual care plus screening and indication, usual care+) with regard to PTSS (primary goal).
2. To implement and evaluate the preventive trauma-focused group intervention ‘Mein Weg’ delivered by trained and supervised social workers for UYRs with subclinical PTSS in CYWS facilities.
3. To implement and evaluate an evidence-based treatment (TF-CBT) in clinical practice and specific TF-CBT training for interpreters.
4. To assess the costs of care for UYRs and the cost utility of the BETTER CARE model in comparison to usual care+.
5. To assess the readiness in CYWS facilities to collaborate with mental health services and the role of context factors in long-term developmental trajectories.

### Trial design

**Fig. 2:**
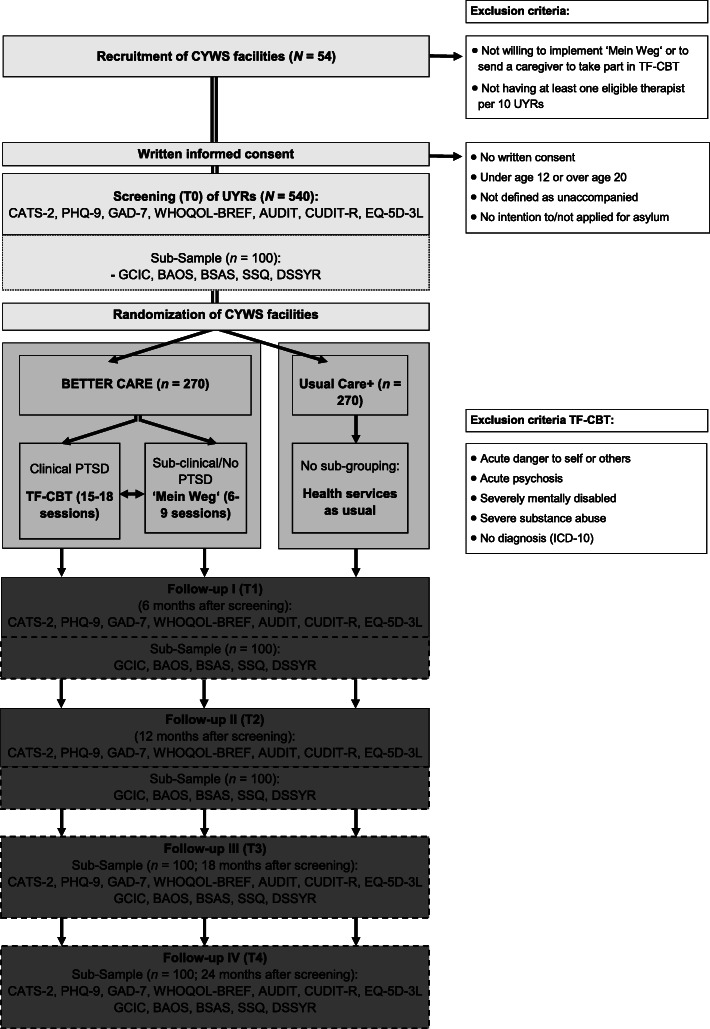
Participant flow. AUDIT = Alcohol Use Disorder Identification Test; BAOS = Brief Acculturation Orientation Scale; BSAS = Brief Sociocultural Adaptation Scale; CATS-2 = Child and Adolescent Trauma Screen 2; CUDIT-R = Cannabis Use Disorder Identification Test, Revised; CYWS = Child and youth welfare service; DSSYR = Daily Stressors Scale for Young Refugees; GAD-7 = Generalized Anxiety Disorder 7; GCIC = Group Climate Instrument for Children; PHQ-9 = Patient Health Questionnaire 9; SSQ = Social Support Questionnaire; TF-CBT = trauma-focused cognitive behavioral therapy; UYR = unaccompanied young refugee; WHOQOL-BREF = World Health Organization Quality of Life, brief version

The current study is a cluster randomized controlled hybrid implementation-effectiveness trial with two active conditions (BETTER CARE versus usual care+). An independent institution (Institute of Epidemiology and Medical Biometry, Ulm University) will perform the randomization of clusters (CYWS facility and one therapist for 10 UYRs), stratified by the number of UYRs in the respective CYWS facility (≤ 20 UYRs = small, > 20 UYRs = big facility). Randomization will be done as stratified block randomization with permuted block length using the randomization software ROM [25]. Clusters will be randomized in either BETTER CARE or usual care+ after agreement to participate in the study, after attendance by CYWS staff in the initial information workshop, and after screening (T0) of participants of the respective facility is completed. Selection bias will be avoided by the large number of clusters (*N* = 54). See Fig. 2 for participant flow through the study.

The study protocol was written in accordance with the SPIRIT 2013 statement (Standard Protocol Items: Recommendations for Interventional Trials; for the SPIRIT Checklist, see Additional file 1).

### Study setting and recruitment

As this is a dissemination and implementation trial, assessment and intervention will take place in a naturalistic setting: the CYWS facilities in which the UYRs live, private practices, and outpatient clinics. Researchers from university clinics will be involved in instructing screenings and implementing the interventions (workshops, case consultation). However, they will not be involved in providing group or individual treatments themselves.

Recruitment will last 24 months and will focus on CYWS facilities serving UYRs and not on UYRs themselves. We will use the following recruitment strategies: letters of invitation to the CYWS, half-day symposia for CYWS staff (presentation of previous findings, the study, and its components), provision of flyers for youth welfare offices and central authorities as well as non-statutory agencies. To recruit therapists, we will publish papers in widely distributed journals for German mental health professionals, and we will send out information by e-mail through professional organizations and personalized information letters. General additional recruitment efforts will include a study website, press interviews, distribution of flyers, and talks at mental health and/or refugee care events.

### Trial status

This study protocol is version 1 from July 4, 2019. Recruitment of CYWS facilities began on July 13, 2020, and is scheduled to be completed on January 1, 2022.

### Participants and eligibility criteria

#### Criteria for CYWS facilities

Inclusion criteria for CYWS facilities are (1) responsible for a number of UYRs of whom at least 8 UYRs have declared an interest in participation; (2) willing to participate in training and implementation of screenings and documentation; (3) agree to name a mental health coordinator (MHC) from their staff; (4) in the case of randomization to BETTER CARE, willing to implement “Mein Weg”; (5) agree to send a caregiver to participate in TF-CBT; and (6) availability of at least one eligible therapist for 10 UYRs ≤ 1 h travel time.

#### Criteria for participants

Inclusion criteria for participants are (1) age 12–20 years; (2) arrived in Germany as unaccompanied minors; (3) applied for asylum or intend to do so; (4) being cared for by a CYWS facility at screening; (5) written informed consent given by participant and legal guardian (if < 16 years at screening); and (6) reported at least one traumatic event in line with the DSM-5 A criterion. No exclusion criteria have been defined for participants in the RCT.

If they are to be referred to and treated with TF-CBT in the event of randomization to BETTER CARE, UYRs have to meet the following criteria: (1) a score (self-report) of ≥ 25 points in the initial screening with the Child and Adolescent Trauma Screen, second version (CATS-2) [26]; and (2) diagnosis of PTSD (according to International Classification of Diseases 10 [ICD-10]) or any mental disorder (ICD-10) as confirmed by the therapist. Exclusion criteria for TF-CBT treatment are (1) acute danger to themselves or others; (2) acute psychosis; (3) acute suicidality; (4) severe substance abuse; or (5) severe mental disability (each as judged by the therapist). Study participants who were excluded from the study will be referred to a more appropriate treatment for their needs, such as inpatient mental health care institutions.

#### Criteria for therapists

Therapists need to meet the following inclusion criteria to participate: (1) licensed as either child and adolescent psychotherapist or psychological psychotherapist or child and adolescent psychiatrist; (2) willingness to participate (i.e., motivation to learn TF-CBT and to invest additional time into training and documentation); (3) written informed consent; and (4) willingness to treat up to 3 UYRs with PTSD. There are no exclusion criteria.

### Sample size

The sample size calculation was based on a two-step procedure due to the hierarchical data structure. First, we calculated the sample size for an individual randomized study based on our previous data. We assumed a small effect size (ES) of *d* = 0.2 for “Mein Weg” as this intervention is implemented with a non-clinical sample and a medium ES of *d* = 0.5 for TF-CBT. The latter ES reflects the fact that our previous trial with *d* = 1.08 (CATS [19]) was uncontrolled, and treatment was provided in a university setting with highly experienced therapists. We plan to compare BETTER CARE with usual care+ and expect a small effect (*d* ≥ 0.30) in favor of BETTER CARE at 12 months (T2) on the CATS-2. A power analysis for a t-test with independent samples (α = 0.05, two-tailed, power 0.80) results in a minimum sample size of 2 × 176 participants (352 in total). The second step will factor in the cluster structure of the data with participants nested within CYWS facilities/therapists clusters. We assume that each CYWS facility will recruit on average *n* = 10 participants. The design effect is needed for the inflation of the group sample size: DE = 1 + (*m* − 1) × ICC with the fixed cluster size *m* and the intra-cluster correlation coefficient ICC. For the fixed cluster size, a value of *m* = 8 was set because of an estimated dropout rate of 20% of participants (UYRs). For the ICC, a liberal value of 0.05 was assumed to control for the correlation of the outcome of participants living in the same facility. In this context, the DE is 1 + (8–1) × 0.05 = 1.35 which results in a group size of 352 × 1.35 = 476 participants. As every facility recruits 10 participants on average, 48 facilities are needed for the whole trial. With an assumed dropout rate of 10% of facilities, an oversampling of 6 facilities is needed, resulting in 54 facilities (= 2 × 27 facilities). Thus, the overall participant sample within these facilities is estimated as 54 × 10 = 540 participants.

### Procedure

Once a CYWS facility has declared its interest in participating in the study, MHCs will be informed about the study details, eligibility criteria, informed consent, and randomization procedure in an initial information workshop. They will also be informed about participant inclusion criteria to ensure that all the participants who are willing to participate are indeed eligible. Eligibility of a facility will be evaluated using a facility questionnaire which will be completed online in the context of a nationwide CYWS survey which is part of the study. Once all facility inclusion criteria are clear, the head of the facility and all principal investigators (RR, HK, RK, UZ) will sign a cooperation agreement.

**Fig. 3:**
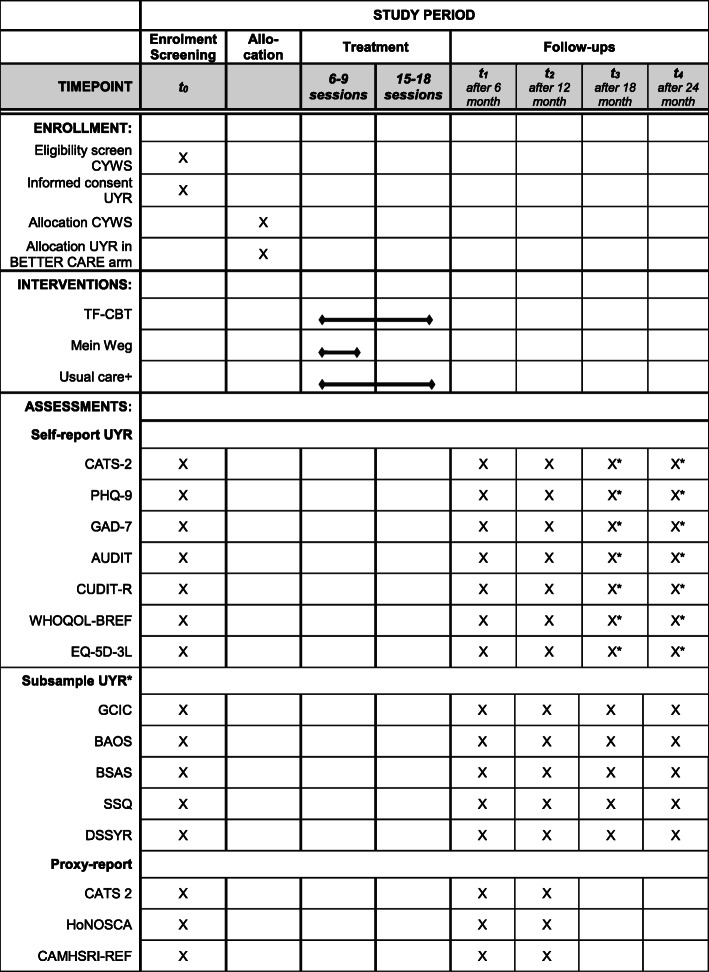
Schedule of enrollment, interventions, and assessments (SPIRIT Figure). AUDIT = Alcohol Use Disorder Identification Test; BAOS = Brief Acculturation Orientation Scale; BSAS = Brief Sociocultural Adaptation Scale; CAMHSRI-REF = Child and Adolescent Mental Health Service Receipt Inventory adapted for refugee youths; CATS-2 = Child and Adolescent Trauma Screen 2; CUDIT-R = Cannabis Use Disorder Identification Test, Revised; CYWS = Child and youth welfare service; DSSYR = Daily Stressors Scale for Young Refugees; GAD-7 = Generalized Anxiety Disorder 7; GCIC = Group Climate Instrument for Children; HoNOSCA = Health of the Nation Outcome Scales for Children and Adolescents; PHQ-9 = Patient Health Questionnaire 9; SSQ = Social Support Questionnaire; TF-CBT = trauma-focused cognitive behavioral therapy; UYR = unaccompanied young refugee; WHOQOL-BREF = World Health Organization Quality of Life, brief version. *Subsample of *n* = 100

Baseline screening (T0) will be undertaken at the respective CYWS facility by study staff and MHCs. All participants will complete self-report questionnaires on an iPad. Proxy-report questionnaires will be completed by the respective caregiver of a participant. After screening, the study center will communicate the result of the subsequent randomization to the facility by letter and telephone. In a next step, each participant will receive a short written report documenting the main findings from the screening to be shared with the caregiver. Follow-up assessments will take place for both study arms, 6 (T1) and 12 months (T2) after randomization (see Fig. 3 for all assessment details). It will also be followed by a short written report on the assessment results. A sub-sample of *n* = 100 participants will be followed up for two further assessments at 18 (T3) and 24 months (T4) after randomization. They will complete additional questionnaires on group climate, social support, and post-migration factors at each time point (T0–T4, see the “Measures” section).

### Interventions

Before randomization, the first step in all participating CYWS facilities will be a systematic screening of psychopathology, psychosocial difficulties, and indication for treatment (PTSS as measured with the questionnaire CATS-2 as the primary outcome (see the “Measures” section)). The MHCs will undergo training in trauma-informed assessment (screening) and will learn how the written report is to be interpreted and how they can derive the individual indication for prevention or psychotherapy in each of their UYRs (indication).

#### BETTER CARE arm

The stepped-care approach BETTER CARE includes the first step as described above (screening and indication). The second step refers to subclinical cases (CATS-2 score < 25). They will be allocated to the preventive group intervention “Mein Weg.” Participants above the clinical cut-off (≥ 25) will be referred to TF-CBT (step 3). UYRs with a CATS-2 score ≥ 25 who refuse to participate in TF-CBT or who have to wait more than 3 months for TF-CBT treatment will be invited to participate in “Mein Weg” (see Fig. 1).

##### Trauma-focused preventive group intervention “Mein Weg”

The manualized intervention can be delivered by trained non-specialist social workers. It empowers young refugees to cope with their traumatic experiences and stress-related symptoms. The rationale of this intervention is derived from the framework of exposure-based and cognitive interventions for trauma-exposed minors. It aims to empower the social workers in the CYWS programs to gain a deeper understanding of the UYRs’ individual (trauma) history and cultural background and to provide appropriate support to the young refugees. “Mein Weg” is a group intervention delivered in 6–8 sessions lasting between 90 and 120 min. It comprises psychoeducation to normalize psychological symptoms, teaching of a relaxation technique to reduce hyperarousal symptoms, a trauma narrative to counteract dysfunctional avoidance and unwanted intrusions of traumatic memories, and cognitive restructuring to reduce trauma-related cognitive distortions. The aims here are to foster social support, help participants regain a sense of safety, and anticipation of self-efficacy with future challenges. In addition to the evaluated “Mein Weg” manual [14], the author will add 2–3 sessions on problem-management strategies related to asylum stressors.

*Implementation of “Mein Weg”*. In order to implement “Mein Weg” within BETTER CARE facilities, social workers in the CYWS facilities will attend a 2-day training in trauma, trauma-related disorders, their treatment, and the implementation of “Mein Weg” by the author of the manual (EP) and certified “Mein Weg” trainers. During the implementation of the program, social workers will receive weekly case consultations and complete session checklists as fidelity checks.

##### Trauma-focused cognitive behavioral therapy

TF-CBT is a short-term, evidence-based treatment for children and adolescents with PTSD which can be delivered in 12 to 15 sessions [17]. It has nine components that are subsumed with the acronym PRACTICE: psychoeducation and parenting skills (components 1 and 2), relaxation, affect modulation, cognitive coping, trauma narrative, in vivo exposure, conjoint parent-child sessions, and enhancement of safety and development. We will extend TF-CBT by three sessions (up to 18 sessions) that will focus on relapse prevention and problem management related to asylum stressors. A trusted caregiver is to be involved in up to 50% of the treatment sessions and the treatment is usually provided in double sessions (one session with the child and one with the caregiver).

*Implementation of TF-CBT*. TF-CBT is specifically suited to improving social support. It requires a lower level of language skills than interventions for adults. Furthermore, as it has been studied with participants aged three to 18, the level of language requirements can be modified to suit the individual patient. Hence, limited language skills or the involvement of interpreters are no barriers to receiving TF-CBT within this trial. The TF-CBT training package for therapists consists of online training (enriched with culturally adapted materials, http://tfkvt.ku.de), an instructor-led 2-day workshop on the treatment, and case consultations on ongoing cases via telephone (biweekly), each provided by certified TF-CBT trainers. The specific aspects of working with UYRs taught in the workshop will be integrated into the “regular” TF-CBT components (i.e., working with interpreters, preparation for future stressors). Therapists will complete the Brief Practice Checklist Revised (BPC-R [27]) after each session as a measure of fidelity. A TF-CBT treatment case will be deemed to have been completed if at least 8 sessions, including a trauma narrative, have been completed.

To further support the implementation of and decrease barriers to ESTs for UYRs, we will develop a 1-day training session on translation in trauma-focused treatments for interpreters that will be proposed to all suitable (lay) interpreters in the communities of clusters randomized to BETTER CARE.

#### Usual care+ arm

The control condition will be called usual care+ as it presents the usual care that UYRs receive in the CYWS facilities and mental healthcare system enhanced with screening and indication procedures offered by study participation (see step 1). All mental health care utilization will be documented for each participant at every assessment so as to be able to offer a clear description of usual care+ afterwards (Child and Adolescent Mental Health Service Receipt Inventory [CAMHSRI-DE]; [28], see the “Measures” section).

### Measures

All self-report measures will be available in the following languages: German, English, French, Arabic, Kurdish, Dari, Farsi, Pashto, Somali, and Tigrinya. They will be recorded on a tablet computer or on paper by the participant. The respective caregiver will respond to the proxy-report measures in German.

#### Primary outcome

The CATS-2 [26] at the 12-month follow-up is the primary outcome. The first part consists of an event checklist with 15 items to be answered yes/no. The second part consists of 20 items corresponding directly to PTSD criteria according to the Diagnostic and Statistical Manual of Mental Disorders 5 (DSM-5 [29]) They measure the severity of PTSS employing a 4-point scale ranging from 0 (never) to 3 (almost always). To evaluate psychosocial functioning, five dichotomous items (yes/no) investigate whether the rated PTSS interfere with key areas of functioning. Good internal consistency (*α* = 0.75–0.93) has been confirmed in the initial validation [26] and in an RCT with UYRs (*α* = 0.75) [12]. At present, the CATS is accessible in several languages and has been successfully used in previous studies with UYRs [2, 19]. For the current study, a slightly adapted version (CATS-2) will be used to cover ICD-11 PTSD and complex PTSD criteria in addition.

#### Secondary outcomes

All secondary outcomes will be evaluated at the 12-month follow-up. The Patient Health Questionnaire (PHQ-9) [30] comprises 9 items that reflect the criteria for the diagnosis of depressive disorders according to DSM-IV. It showed good reliability (*α* = 0.86–0.89) and has been validated in many contexts and languages [30, 31].

The Generalized Anxiety Disorder Assessment (GAD-7) [32] comprising 7 items assesses symptoms of anxiety according to DSM-IV and has been validated (*α* = 0.92) in many contexts and languages [31].

The World Health Organization Quality of Life-BREF (WHOQOL-BREF [33]) comprises 26 items measuring 4 key areas (physical health, psychological health, social relationships, and environment) over the previous 4 weeks [33]. The short version has been validated in many languages. The Cronbach’s *α* tested in international samples was 0.68–0.82 [34].

#### Exploratory outcomes

The primary and secondary outcomes will be analyzed in an exploratory manner at the 6-month follow-up.

The Alcohol Use Disorders Identification Test (AUDIT) is a 10-item questionnaire used to assess recent excessive drinking and is in conformity with ICD-10 definitions [35]. It is a well-validated (α = 0.80–0.94) [36] measure of risk across gender, age, and cultures.

The Cannabis Use Disorder Identification Test, Revised (CUDIT-R) is an 8-item questionnaire used to assess cannabis use disorder severity [37]. The CUDIT-R has been validated (*α* = 0.91) as both a screening test and a rating scale for cannabis use disorder severity [37].

The EQ-5D-3L [38] is a two-part instrument for the definition and the valuation of health states as the basis for the estimation of quality-adjusted life years (QALYs). It consists of a descriptive part and a visual analog scale similar to a thermometer [39]. At present, the EQ-5D-3L is available in more than 180 languages including all those expected to be relevant for the project.

#### Proxy measures

The following measures will be detailed by the respective caregiver of each participant: The CATS-2 caregiver version [26] which comprises the same items as the CATS self-report with a reference to the child/adolescent (“has your child…”).

One item derived from the Nation Outcome Scales for Children and Adolescents (HoNOSCA) [28] will be used to obtain a proxy estimation of substance abuse.

The CAMHSRI-DE [28] is an interview tool for the assessment of medical and psychosocial services used by children and adolescents with mental health problems based on the European version CAMHSRI-EU [40]. Service costs will be estimated by combining service use assessed by the CAMHSRI-DE with unit costs gathered from a broad spectrum of information sources. The CAMHSRI-DE will be adapted for refugee youths (CAMHSRI-REF) and will be administered by the caregiver in collaboration with the UYR.

#### Long-term follow-up sub-sample

A subsample of *n* = 100 will be assessed with additional questionnaires and will be followed up for 18 (T3) and 24 months (T4). The short version of the Group Climate Instrument for Children (GCIC) [41] consisting of 14 items assessing open and closed climate in institutions will be used. This questionnaire showed good to moderate reliability for the open (Cronbach’s *α* = 0.91) and the closed scales (Cronbach’s *α* = 0.71). It has been validated in the context of residential care in different countries [41, 42].

The Brief Acculturation Orientation Scale (BAOS) [43] is a bidimensional instrument assessing the acculturation orientation towards the home country and the host country independently. The home and host country factors of the BAOS showed acceptable reliability (Cronbach’s *α* = 0.72–0.78).

The Brief Sociocultural Adaptation Scale (BSAS) [43] is a 12-item questionnaire that assesses various aspects of sociocultural adaption (e.g., language, climate, people, values, and beliefs). The scale demonstrated good reliability (Cronbach’s *α* = 0.85); it is available and has been validated in different languages.

The questions on social support are based on the Social Support Questionnaire (SSQ6-G) [44]. In its original version, the three scales showed good reliability (Cronbach’s *α* = 0.71 to 0.92).

The Daily Stressors Scale for Young Refugees (DSSYR) [45] examines the extent to which 15 different post-migration daily stressors were experienced by the participants during the previous month.

#### Measures for “Mein Weg” trainers and TF-CBT therapists

Both groups of professionals will complete questionnaires before and after the training (therapists also before the online training) as well as 6 and 12 months post-training. The Professional Quality of Life (ProQOL) is a 30-item questionnaire that measures the negative and positive effects of helping others who experience suffering and trauma [46]. The items form 3 subscales corresponding to compassion satisfaction (*α* = 0.89–0.91), burnout (*α* = 0.74–0.80), and secondary traumatic stress (*α* = 0.86–0.89) [47].

The Evidence-Based Practice Attitude Scale (EBPAS-36) is a 36-item questionnaire derived from the EBPAS-50. It assesses the attitudes of mental health providers towards the adoption of evidence-based practice [48]. The measure has been well validated [48].

As “Mein Weg” trainers also work as professionals in CYWS facilities, they will be asked to complete the Implementation Climate Scale (ICS). It measures the degree to which the organizational climate is supportive of evidence-based practice implementation [49]. High internal consistency has been confirmed for the total scale (*α* = 0.91), and for the subscales (*α* = 0.81–0.91).

#### Measures for translators

All participants in the TF-CBT specific translator training will complete measures at four time points: before and after training and 6 and 12 months post-training. They include an evaluation of the translator training (14 items), experiences and attitudes towards translating in mental health contexts (41 items), and the PTSD Checklist for DSM-5 (PCL-5) [50].

### Methods against bias

Random allocation of facilities and participants is ensured by the trial design. All training sessions will be highly standardized and treatment fidelity will be ensured by ongoing case consultations and by session checklists in both interventions. The blinding of UYRs is not possible. All outcomes on the patient level will be well-established measures. Data records will be kept as up-to-date as possible by means of online symptom monitoring and documentation of treatment sessions.

### Data management and storage

Data will be directly entered in an online assessment tool by the relevant informant. Data collected using the paper-pencil procedure will be entered in the same database by an independent data manager. As almost all data will be directly entered in the database, no source data verification will be needed. Incoming data will be continuously monitored and checked for plausibility, quality, and completeness by an independent data manager installed at the University Clinic Ulm. All data will be stored and transferred in pseudonymized form. All the main participants and the IT company responsible for the assessment tool of the trial have signed and followed a data protection concept in accordance with the General Data Protection Regulation (EU) 2016/679 (GDPR). Only authorized and trained study personnel will receive a login-roll in line with their task (e.g., research assistant to start screening, data manager for query management, data entry, data validation, and plausibility checks). For the purpose of long-term storage, the original, pseudonymized data (after database lock) will be stored at the University Clinic Ulm and with the relevant project partners. They will be made available on request to scientific colleagues after the publication of the results.

### Statistical analysis

We will analyze the primary outcome (CATS-2 severity score) using hierarchical linear mixed (HLM) models to account for the nesting of cases within clusters. Mixed effect models, with fixed effects of group (BETTER CARE vs usual care+) and time (intake (T0) versus 6 months (T1) versus 12 months (T2)) as well as their interaction, will be conducted. Primary outcome analysis will be based on the ITT sample (*N* = 540 participants) as the mixed model approach will take missing data and different numbers of reassessments per participant/patient into account. A significance level of *α* = 0.05 will be used. We will also use exploratory mixed linear models to examine the impact of other variables (gender, age, nationality, asylum status) on our primary outcome. Separate (hierarchical) linear mixed models will be used to analyze the secondary outcomes.

### Ethical considerations, monitoring, and safety

The study was planned and will be conducted in accordance with the International Council for Harmonization Guideline for Good Clinical Practice [51]. It has been approved by the ethics review board of the University Ulm (07 October 2019) and Eichstaett-Ingolstadt (08 November 2019). The trial is registered under German Clinical Trials Register (www.germanctr.de; registration number DRKS00017453, date 11 December 2019).

Study safety will be ensured by monitoring for the incidence of serious adverse events (SAEs; e.g., suicide attempts, unplanned hospitalizations, occurrence of life-threatening conditions) at all follow-up assessments. All such incidents and other aspects of study safety will be regularly reported to an independent Data and Safety Monitoring Board, which offers advice on protocol changes in the event of such incidences, or even on discontinuation of the trial. However, no harm to participants is to be expected. In our previous studies with UYRs, we did not detect any harm arising from the interventions for the participants [12, 19].

All participants will be informed about the study in oral and written form, with details of the trial’s procedures, risks, costs, confidentiality, data storage, and about the right to discontinue participation at any time without giving any reasons. Participants will be free to continue treatment with their therapists when they quit the research program. Written informed consent will be obtained from participants and, in case of the minors, from their parents/legal guardians. Furthermore, caregivers, “Mein Weg” trainers, psychotherapists, and translators will give their written informed consent. In general, treatment and the associated processes of change and diagnostic procedures may be evaluated as stressful by the participant [52]. Therefore, symptoms may temporarily worsen. In the context of TF-CBT and “Mein Weg,” the development of a trauma narrative can be experienced as stressful as it involves direct confrontation with the traumatic experience and avoided stimuli. However, participants can directly benefit from successful treatment and long-term symptom reduction can be achieved. Even for the control group who will not receive the stepped-care, we do not expect any harm: in two previous studies [2, 8], we assessed young refugees’ mental health without providing further interventions. No serious or adverse events were reported during the two studies.

The study offers the following incentives/compensations: CYWS facilities will receive a lump sum per case for each participant at randomization (€60). Participants will receive compensation for taking part in assessments (€30 each). They may receive compensation for the costs of travel to study therapists of up to €10 per session. In addition, caregivers from CYWS will receive compensation of €40/session for participating in TF-CBT sessions. Therapists and interpreters will receive compensation for travel costs to the workshops (up to €100/person). In addition, therapists may receive compensation for participation in case consultations (€30/h) and for additional diagnostic and documenting activities (up to €240/case). In the event that treatment or translation costs are not covered, costs for one third of treatment or translation cases can be covered by study funds.

## Discussion

This study will be the first hybrid RCT on the effectiveness and implementation of a stepped-care approach for UYRs with PTSS. In addition, it will be the first to test ESTs in a stepped-care approach for this target group. As UYRs seldom receive ESTs or any preventive or psychotherapeutic treatment at all, it is of the utmost importance (1) to create, implement, and disseminate effective and cost-economic treatments and (2) to find ways to allocate individual UYRs, in line with their needs and available resources, to the treatment with the best fit. The study aims to report not only on the effectiveness of these interventions, but also on their implementation in routine care, outside the university setting. In this context, system and organizational barriers will also become visible, which can hinder the successful implementation process in the German welfare system. We believe that the transfer of knowledge and skills after the end of the BETTER CARE project will be increased. We hope to create a mental health landscape that is open and qualified for the needs of UYRs.

### Dissemination

Dissemination will be carried out on several levels. Study results will be published within a scientific frame, via publications, conferences, and talks. The results will also be shared with participants, CYWS staff, and political and societal stakeholders. In addition, we will be able to approach politicians and stakeholders in the health care system by sharing findings on the cost-effectiveness of the stepped-care approach and the usual care in German CYWS. Since this projects aims to reach many CYWS facilities, knowledge on trauma, trauma-related disorders, and how to treat them will be disseminated in many parts of southern Germany throughout different professions (social workers, therapists, translators). The sustainable implementation of trauma-focused interventions will enable trained personnel to implement trauma-informed care not only for study participants but also for non-participants (with and without a migration background). By participating in BETTER CARE, the CYWS system will be empowered on several levels and could then serve as a blueprint for further implementation efforts. To the extent that data are de-identifiable, we will make them available in accordance with IRB.

### Strengths and limitations

This study has several strengths: (1) By offering screenings in 10 different languages and by waiving exclusion criteria for UYRs, we will endeavor to recruit a sample that is as representative as possible. (2) By assessing all the variables and the additional assessment of post-migration factors in a subsample, we will be able to analyze predictors (mediators and moderators) of the approach’s effectiveness. (3) The project is aware of the challenges in the CYWS (e.g., staff turnover) and will seek to determine the facilities’ readiness to collaborate with mental health services. Furthermore, the study aims to empower the CYWS by means of training in screening and “Mein Weg.” (4) By randomizing facilities and using the control condition usual care+ where we document all (mental) health care offers to the participants, we aim to furnish reliable evidence of the actual care for UYRs which can then be compared to BETTER CARE. (5) This study claims a high external validity that represents the natural mental health and child welfare context in which UYRs in Germany normally reside.

Possible limitations to the trial are as follows: As we provide the measures in ten languages, some language versions are newly translated and have not yet been validated. In addition, we will not be able to cover all the languages of the UYR population in Germany. The assessment only includes questionnaires. We will not, therefore, be able to report on clinical diagnoses except for the subgroup receiving TF-CBT and, hence, receiving diagnoses by the participating therapists. As we recruit and randomize CYWS facilities, we will not be able to reach UYRs living with foster families or accompanied young refugees living with their parents. Furthermore, asylum applications in Germany have been declining in the last years [53] and a decreased number of UYRs in southern Germany could complicate recruitment. We believe that the CYWS facilities participating in the trial will show a high motivation to improve mental health care for UYRs that is probably beyond average. Additionally, therapists willing to participate may be particularly interested in providing ESTs or in working with UYRs. As they will be randomized in clusters, even the CYWS facilities in usual care+ may have highly motivated therapists in the vicinity. This could increase the options for referring UYRs to treatment. However, we will also face unknown system, organizational, and implementation barriers. This may be because stepped-care models have often been suggested recently but have not been extensively studied in the German mental health care system.

Results from the BETTER CARE trial should provide information that will be beneficial for practitioners, organizations, and researchers in the field of dissemination and implementation, and policy makers looking for knowledge on how to implement needs-adapted stepped-care approaches for vulnerable populations who face considerable barriers to adequate treatment.

### Trial status

At the time of manuscript submission (June 2020) the study had started. We are in the early stages of information workshops for CYWS staff. The implementation of screenings has not started due to restrictions caused by the COVID-19 pandemic. So far, *n* = 8 CYWS facilities have been recruited but not randomized and *n* = 0 UYRs have been screened.

## Supplementary Information

**Additional file 1.** SPIRIT 2013 Checklist: Recommended items to address in a clinical trial protocol and related documents.

## Data Availability

The dataset(s) supporting the conclusions of this article are available upon request from authors.

## Abbreviations

AUDIT: Alcohol Use Disorders Identification Test
BAOS: Brief Acculturation Orientation Scale
BSAS: Brief Sociocultural Adaptation Scale
CATS: Child and Adolescent Trauma Screen
CUDIT-R: Cannabis Use Disorder Identification Test, Revised
CYWS: Child and youth welfare service
DSM-5: Diagnostic and Statistical Manual of Mental Disorders, 5th edition
DSSYR: Daily Stressors Scale for Young Refugees
EBPAS: Evidence-Based Practice Attitude Scale
ES: Effect size
ESTs: Empirically supported treatments
GAD-7: Generalized Anxiety Disorder Assessment
GCIC: Group Climate Instrument for Children
HoNOSCA: Nation Outcome Scales for Children and Adolescents
ICD-10: International Classification of Diseases
ICS: Implementation Climate Scale
MHCs: Mental health coordinators
ProQOL: Professional Quality of Life
PTSD: Posttraumatic stress disorder
PTSS: Posttraumatic stress symptoms
RCT: Randomized controlled trial
SSQ6-G: Social Support Questionnaire
TF-CBT: Trauma-focused cognitive behavioral therapy
UYRs: Unaccompanied young refugees
